# An Investment in Images

**DOI:** 10.19102/icrm.2017.080305

**Published:** 2017-03-15

**Authors:** Claudio Tondo


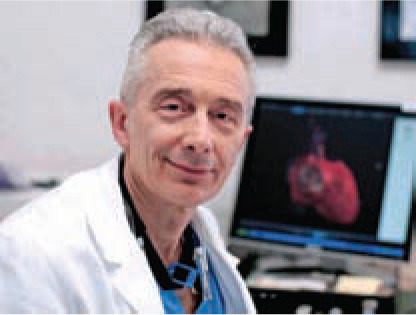


I wish to take the opportunity to introduce the newest section of the *Journal of Innovations in Cardiac Rhythm Management,* “Cardiac Images in EP,” which I have the honor to chair. We are witnessing wonderful and fast advancements in the field of arrhythmia research, and, surely, images that come from either ECG tracings, vascular pictures or even ultrasound, CT-scan or cardiac-MRI have already greatly improved our understanding of cardiac electrophysiology (EP) and its relationship with human anatomy.

I believe that this new section of the *Journal* will provide everyone with the chance to share specific EP images with many of us and thus, expand our knowledge and further our understanding of different clinical scenarios. Therefore, this section should be viewed as an opportunity to shed new light on different topics and to enjoy challenging cases.

I therefore welcome all of you to send in your best EP images each month in order to share your interpretation with us and the readers. I also invite the readers to comment on each case, and to provide alternative explanations as to ignite the discussion. Additionally, I suggest the inclusion of a brief two to four paragraphs to accompany the image, to provide background and/or explanation as to the rationale behind the interpretation.

I look forward to receiving interesting materials from all of you.

Sincerely,

Claudio Tondo, MD, PhD, FESC

Professor of Cardiology

Chairman, Cardiac Arrhythmia Research Center

Monzino Cardiac Center, IRCCS

Department of Cardiovascular Sciences and Community

University of Milan, Milan, Italy

claudio.tondo@ccfm.it; claudio.tondo@unimi.it

